# The knowledge of primary health care professionals regarding mental health: diagnosis by mhGAP

**DOI:** 10.11606/s1518-8787.2023057005272

**Published:** 2024-04-01

**Authors:** Joana Moscoso Teixeira de Mendonça, Ilana Eshriqui, Leticia Yamawaka de Almeida, Valmir Vanderlei Gomes, Lívia Schunk, Ana Alice Freire de Sousa, Larissa Karollyne de Oliveira Santos, Sandra Fortes

**Affiliations:** I Hospital Israelita Albert Einstein Centro de Estudos, Pesquisa e Prática em Atenção Primária à Saúde e Redes São Paulo SP Brazil Hospital Israelita Albert Einstein. Centro de Estudos, Pesquisa e Prática em Atenção Primária à Saúde e Redes. São Paulo, SP, Brazil; II Universidade do Estado do Rio de Janeiro Departamento de Especialidades Médicas Rio de Janeiro RJ Brazil Universidade do Estado do Rio de Janeiro. Departamento de Especialidades Médicas. Rio de Janeiro, RJ, Brazil

**Keywords:** Mental Health, Primary Health Care, Health Human Resource Training

## Abstract

**OBJECTIVE:**

To analyze knowledge about priority topics in mental health care of strategic actors who work in regions where the Health Care Planning (PAS) methodology is used.

**METHODS:**

This is a quantitative, descriptive, cross-sectional, and observational study carried out with professionals from six health regions, distributed in three Brazilian states (Goiás, Rondônia and Maranhão) and linked to the project “Saúde mental na APS” (Mental health in PHC) of the *Programa de Apoio ao Desenvolvimento Institucional do Sistema Único de Saúde* (Proadi-SUS – Institutional Development Support Program of the Brazilian Unified Health System). The sample was made up of professionals who participated in the intervention guide multiplier training stage for mental, neurological and alcohol and other drug use disorders in the primary health care network, from July to September 2022. Data collection was through a self-administered instrument, in electronic format, consisting of a block with socioeconomic items and a structured questionnaire to assess participants’ knowledge about priority topics in mental health. Descriptive analyses and comparison of proportions were conducted to analyze the data.

**RESULTS:**

A total of 354 health professionals participated in the study. Regarding the percentage of correct answers in the questionnaire on priority topics in mental health, the highest medians were identified in the “Depression” module. On the other hand, the content referring to the modules “Essential care and practices” and “Other important complaints” presented the lowest values. Furthermore, some participant characteristics were found to be associated with the percentage of correct answers in the questionnaire modules.

**CONCLUSIONS:**

The findings reveal opportunities for improvement, mainly in knowledge related to communication skills and the approach to emotional and physical distress without diagnostic criteria for a specific disease, offering support for planning actions aimed at intensifying the consideration of these themes during the operational stages of PAS.

## INTRODUCTION

In the context of mental health care, the growing global burden of mental disorders^1^associated with their great economic burden^2,3^and the scarcity of human resources with adequate knowledge in mental health are elements of a scenario related to the gap between the need for care and the provision of minimally effective treatment for these conditions^4,5^.

The proposal to address this gap advocates, among other aspects, the establishment of an effective care network that encompasses a diverse set of services, since only those specialized in mental health will not guarantee comprehensive care^6^.

Thus, primary health care (PHC) services stand out as strategic in offering mental health care, especially for two reasons: the prevalence of mental disorders^7^and the fundamental role of PHC in tackling highly prevalent health conditions; and comprehensive care, as an attribute of PHC^8^since comprehensive care cannot be offered and mental health issues neglected^9^.

In this sense, the Mental Health Gap Action Programme (mhGAP), launched by the World Health Organization (WHO) in 2008, seeks to answer the question: “what can be done in routine health care, by non-expert health professionals, to treat people with mental disorders?”^10^

Among the developments of this action plan, the WHO prepared the first version of the mhGAP Intervention Guide (mhGAP-IG), which is updated periodically to support the implementation of the proposed guidelines, based on the training of non-expert PHC professionals. Version 2.0 of MhGAP-IG^11^is characterized as a low-cost and high-impact technical tool, which presents the integrated management of mental, neurological, and alcohol and other substance use disorders, through the use of protocols for clinical decision.

It is noteworthy that, in addition to technical knowledge, mental health work in PHC requires professionals to have knowledge about public health policies, the territory, the population’s epidemiological profile and the care network, as well as the skills to welcome, listen, communicate, and work as a team^12^.

In this way, the Support Program for the Institutional Development of the Unified Health System (Proadi-SUS) project, entitled “Mental health in PHC,” combines the training strategy for the use of MhGAP-IG to evaluate, manage and follow up on priority themes in mental health in PHC with the Health Care Planning (PAS) methodology. This methodology allows for improving the competence of healthcare teams in planning, organizing, and monitoring work processes, focusing on the needs of the users under their responsibility^13^.

The process is anchored in a robust pedagogical component, with a view to developing knowledge, skills and attitudes in workers and managers to offer quality health care that adds value to the user^14^. Its objective is to develop teams for adequate operationalization of Health Care Networks (RAS), with an emphasis on several priority lines of care. This union aims to strengthen the line of mental health care, as it qualifies PHC to play its role as organizer and coordinator of health care in the RAS.

Given this context, it is understood that in order to develop strategies for organizing and qualifying mental health care, it is necessary to diagnose the professionals’ level of prior knowledge on the subject. Thus, this study aimed to analyze the knowledge on priority topics of mental health care among higher-level PHC professionals who work in regions that implement the PAS methodology and were nominated to be training multipliers for using the MhGAP-IG.

## METHOD

### Study Setting

The study was carried out in the health regions that participate in the Proadi-SUS Project “Mental health in PHC,*”* namely: three in the state of Goiás, two in Rondônia and one in Maranhão. The regions were selected according to three priority criteria: 1) prior experience of at least two years in PAS implementation; 2) macro-processes of the “Social Construction of PHC,” with improvement cycles initiated (e.g.: situational diagnosis, territorialization, family registration, among others); and 3) coverage of municipalities implementing PAS. It should be noted that the “Mental Health in PHC*”* project does not aim to implement PAS in these regions, but to qualify the processes already implemented (or under implementation), focusing on mental health care-related issues.

The Figure shows the operational design of the “Mental health in PHC” project, in which the training of multipliers and professionals to use the MhGAP-IG is carried out, in a process transversal to the execution of the PAS.

**Figure:**
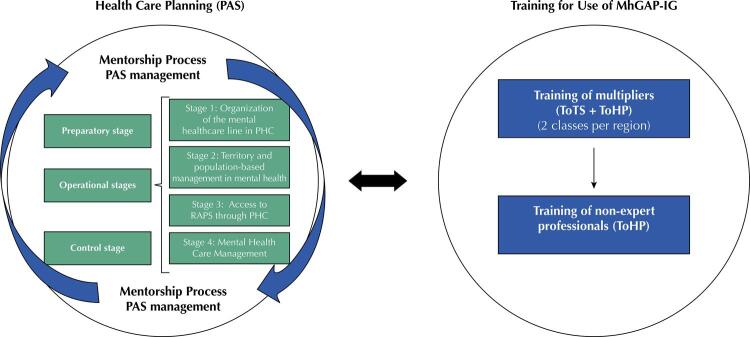
Operational design of the “Mental health in PHC” project.

PHC: Primary health care; RAPS: Psychosocial Care Network; ToHP: Training of health professionals; ToTS: Training of trainers and supervisors; mhGAP-IG: mhGAP Intervention Guide (mental health Gap Action Programme)

As can be seen, in the context of PAS, management actions and mentoring processes are carried out, in light of Plan-Do-Study-Act (PDSA)^15^continuous improvement cycles, in order to qualify work processes throughout the themes addressed in the four operational stages. Through PAS management, support is provided to the technical staff of the State Health Departments (SES) and Municipal Health Departments (SMS) for decision-making throughout the preparatory, operational and control stages. From the mentoring process, moments of conceptual theoretical alignment, reflection, planning, and execution of improvements are carried out, so that changes can be made in the *modus operandi* of teams and services^16^.

Training on the use of MhGAP-IG is carried out transversally to the PAS. This stage is organized in two stages, as recommended by the WHO^17^:

- Training of health professionals (ToHP), which seeks to encourage the development of essential skills for the care of people with mental, neurological and substance use (MNS conditions) disorders, which, in the present proposal, involve the modules practical and essential care; depression, self-aggression and suicide, psychosis, other significant mental health complaints and problematic alcohol use)^17^
- Training of trainers and supervisors (ToTS), which aims to prepare professionals (multipliers) responsible for replicating content in their regions, based on the presentation of proposals and teaching-learning models of adults^17^.

When dealing specifically with the training of multipliers, the training has a workload of 40 hours, distributed between ToHP and ToTS activities. In order to facilitate the organization of multiplication in different territories, participants were divided into two groups. Class “A” trained in the “Depression” and “Self-aggression and Suicide” modules, and class “B” trained in the “Psychosis” and “Problematic use of alcohol” modules.

Furthermore, the “Essential care and practices” and “Other important complaints” modules were covered in both classes, as it is understood that they deal with basic aspects of care and are routine work in mental health in PHC. At the end of the training, pairs were formed with representatives from classes A and B, to conduct the multiplication of the six ToHP modules to other PHC professionals from the municipalities in the participating regions.

### Study Design

This is a quantitative, descriptive, cross-sectional, observational study approved by the Research Ethics Committee of Hospital Israelita Albert Einstein (opinion number: CAAE 12395919000000071).

### Population and Study Sample

Sampling was by convenience, considering the health professionals indicated to participate in the MhGAP-IG multiplier training. The nomination was based on the following criteria, suggested by the project team: higher education in the health area; professional experience in the area of mental health in PHC; working in state and municipal departments or in health services in the aforementioned regions, presenting pedagogical and communication skills and having available workload to carry out the multiplication of training in the regions.

Thus, for this study, the 366 professionals who participated in the multiplier training from July to September 2022 were considered eligible. Of these, 197 completed the activities in class “A,” 125 in class “B” and 44 completed the training in both classes. It should be noted that, among eligible professionals, only 83 mentioned having training in mental health.

### Recruitment and Data Collection

The professionals were approached according to the schedule of the multiplier training classes (July to September 2022), so that the invitation to participate in the study and data collection were carried out before the training began. On that occasion, the main aspects of the research were presented and an invitation to participate in the study was made. The Free and Informed Consent Form was made available in electronic format and those who expressed interest in participating in the study confirmed acceptance by signing the document and were then directed to a self-administered structured instrument, consisting of a block with sociodemographic and professional items; followed by the mhGAP^17^questionnaire to assess their knowledge about priority topics in mental health, translated into Portuguese by the team responsible for training at the Universidade Estadual do Rio de Janeiro. The questionnaire consists of 50 multiple-choice questions, distributed across five assessment modules, which were applied as planned in classes A and/or B (Chart). It is noteworthy that the training in the “Problematic use of alcohol” module was adapted to the Brazilian culture and, therefore, it was decided not to consider the items relating to this topic in the questionnaire. The scheduled time for answering the questionnaire was 20 minutes.

**Chart:** Description of the content covered in the structured questionnaire (July to September 2022).

Modules	Number of Questionnaire Items	Classes
Essential Care and Practices	15 items	A and B
Other Important Complaints	08 items	A and B
Depression	10 items	A
Self-aggression/Suicide	8 items	A
Psychosis	9 items	B
Problematic Alcohol Use	Not rated	B

The study data was collected and managed using the software Research Electronic Data Capture (REDcap)^18^, which is a platform for collecting, storing and managing research data. In this way, the risk of loss of confidentiality is minimal since the data is hosted and managed only through institutional access to the Redcap platform through a password with a double-layer protection system (login and password).

### Data analysis

Correct answers were coded as 1 and incorrect answers as 0. From this, the grades in each module were calculated, which were obtained from the sum and calculation of the percentages of correct answers per multiplier training module. The percentages of correct answers were described as median and interquartile range and categorized as < 50%; 50–75%; ≥ 75% of correct answers. Frequencies and proportions of the participants’ characteristics were described, as well as the percentages of correct answers. The chi-square test was used to compare the proportions of the correct categories according to participant characteristics. A p-value < 0.05 was adopted to identify statistically significant associations. The Stata software (version 12, 2011, StataCorp LP) was used for data analysis.

## RESULTS

Among the 366 participants in the multiplier training, 354 (96.7%) agreed to participate in the research. Table 1 describes the participants’ characteristics, who were on average 37.6 years old (SD = 10.1) and, for the most part, were female (83.7%) and reported brown skin color (57.6%). There was a predominance of professionals with nursing training (50.5%), graduate studies (*lato sensu*) (57.3%), who have worked in their current position for more than a year (76.5%) and were working in a basic health unit (UBS) (41.0%).

**Table 1:** Sociodemographic and professional profile of participants (July to September 2022).

Characteristics	n (%)
Classes (n = 354)	
A	197 (55.6)
B	138 (39.0)
A and B	19 (5.4)
Sex (n = 345)	
Female	289 (83.7)
Male	56 (16.2)
Race/color (n = 342)	
White	123 (36.0)
Brown	197 (57.6)
Black	21 (6.1)
Yellow	1 (0.3)
Professional category (n = 305)	
Doctor	21 (7.8)
Nurse	154 (50.5)
Psychologist	69 (22.6)
Dental surgeon	12 (3.9)
Social worker	10 (3.3)
Others^a^	36 (11.8)
Education level (n = 342)	
Undergraduate	116 (33.9)
Graduate (*lato sensu*)	196 (57.3)
Graduate (*stricto sensu*)	30 (8.8)
Location of operation (n = 344)	
State Health Department	90 (26.2)
Municipal Health Department	73 (21.2)
Basic health unit	141 (41.0)
Specialty in mental health	16 (4.6)
Others	24 (7.0)
Time working in the position (n = 344)	
< 1 year	81 (23.5)
≥ 1 year and < 5 years	174 (50.6)
≥ 5 years	89 (25.9)
State in which the training was carried out (n = 339)	
Goiás (GO)	169 (49.8)
Maranhão (MA)	68 (20.1)
Rondônia (RO)	102 (30.1)

^a^ Professional categories with a proportion of less than 3% (Physical Education, Nutrition, Speech Therapy, Occupational Therapy, Physiotherapy, Pharmacology, Administration, and related areas).

In relation to prior knowledge on priority mental health topics, assessed by the questionnaires, the highest median percentage of correct answers was identified in the “Depression” module. On the other hand, the content referring to the “Essential care and practices” and “Other important complaints” modules presented the lowest values (Table 2).

**Table 2:** Description of the percentage of correct answers in the modules offered in the training (July to September 2022).

Module	Median (IQR)	% of correct answers		

		< 50%	≥ 50 and < 75%	≥ 75%
		
		n (%)	n (%)	n (%)
Essential care and practices (n = 328)	66.7(53.3–73.3)	44 (13.4)	236 (72.0)	48 (14.6)
Depression (n = 199)	90.0 (80.0–100.0)	1(0.5)	31(15.6)	167(83.9)
Psychosis (n = 143)	77.8 (66.7–88.9)	14 (9.8)	52 (36.4)	77(53.8)
Suicide and self-aggression (n = 199)	75.0 (62.5–87.5)	10 (5.0)	52 (26.1)	137(68.9)
Other important complaints (n = 325)	62.5 (50.0–75.0)	69 (21.2)	157 (48.3)	99(30.5)

IQR: Inter Quartile Range.

Table 3 presents a description of the percentage of correct answers in the training modules, according to the participants’ characteristics. The professional category variable was found to be associated with knowledge regarding the “Essential care and practices” (p = 0.004), “Other important complaints” (p = 0.033), and “Suicide and self-mutilation” (p = 0.044) modules, so that doctors seemed to perform better in the “Essential care and practices” and “Other important complaints” modules; and psychologists and dental surgeons in the “Suicide and self-mutilation” modules.

**Table 3:** Description of the percentage of correct answers in the training modules, according to participants’ characteristics (July to September 2022).

Characteristics	Essential care and practices				Other important complaints				Depression				Psychosis				Suicide and self-aggression			
				
	% of correct answers – n (%)			p-value	% of correct answers – n (%)			p-value	% of correct answers – n (%)			p-value	% of correct answers – n (%)			p-value	% of correct answers – n (%)			p-value
				
	< 50%	≥ 50 and < 75%	≥ 75%		< 50%	≥ 50 and < 75%	≥ 75%		< 50%	≥ 50 and < 75%	≥ 75%		< 50%	≥ 50 and < 75%	≥ 75%		< 50%	≥ 50 and < 75%	≥ 75%	
Professional category																				
Doctor	2 (8.3)	14 (58.3)	8 (33.3)	0.004	5 (20.8)	6 (25.0)	13 (54.2)	0.033	1 (7.1)	2 (14.3)	11 (78.6)	0.064	1 (10.0)	2 (20.0)	7 (70.0)	0.823	2 (14.3)	2 (14.3)	10 (71.4)	0.044
Nurse	15 (10.3)	115 (79.3)	15 (10.3)		35 (24.3)	73 (50.7)	36 (25.0)		0 (0)	19 (20.4)	74 (79.6)		6 (10.3)	23 (39.7)	29 (50.0)		4 (4.3)	31 (33.3)	58 (62.4)	
Psychologist	5 (7.8)	48 (75.0)	11 (17.2)		8 (12.5)	35 (54.7)	21 (32.8)		0 (0)	2 (4.9)	39 (95.1)		2 (6.9)	9 (31.0)	18 (62.1)		0 (0)	5 (12.2)	36 (87.8)	
Dental surgeon	3 (25.0)	6 (50.0)	3 (25.0)		4 (33.3)	4 (33.3)	4 (33.3)		0 (0)	1 (14.3)	6 (85.7)		1 (16.7)	1 (16.7)	4 (66.7)		0 (0)	1 (14.3)	6 (85.7)	
Social worker	1 (11.1)	7 (77.8)	1 (11.1)		0 (0)	5 (55.6)	4 (44.4)		0 (0)	1 (20.0)	4 (80.0)		0 (0)	1 (25.0)	3 (75.0)		1 (20.0)	1 (20.0)	3 (60.0)	
Others^a^	11 (31.4)	19 (54.3)	5 (14.3)		12 (34.3)	15 (42.9)	8 (22.8)		0 (0)	1 (16.7)	20 (80.3)		2 (18.2)	5 (45.4)	4 (36.4)		3 (12.5)	7 (29.2)	14 (58.3)	
Education level																				
Undergraduate	18 (16.7)	83 (76.8)	7 (6.5)	0.029	30 (27.8)	52 (48.1)	26 (24.1)	0.079	0 (0)	14 (20.3)	55 (79.7)	0.013	4 (9.5)	16 (38.1)	22 (52.4)	0.714	6 (8.7)	21 (30.4)	42 (60.9)	0.045
Graduate (*lato sensu*)	20 (10.8)	132 (71.4)	33 (17.8)		33 (17.9)	94 (51.1)	57 (31.0)		0 (0)	15 (13.8)	94 (86.2)		10 (11.6)	29 (33.7)	47 (54.7)		3 (2.8)	30 (27.5)	76 (69.7)	
Graduate (*stricto sensu*)	3 (10.7)	18 (64.3)	7 (25.0)		4 (14.3)	11 (39.3)	13 (46.4)		1 (6.2)	2 (12.5)	13 (81.3)		0 (0)	6 (46.1)	7 (53.9)		1 (6.3)	0 (0)	15 (93.7)	
Place of operation																				
State Health Department	11 (13.4)	60 (73.2)	11 (13.4)	0.332	22 (26.8)	40 (48.8)	20 (24.4)	0.776	0 (0)	9 (20.0)	36 (80.0)	0.081	3 (6.5)	14 (30.4)	29 (63.1)	0.445	2 (4.4)	14 (31.1)	29 (64.4)	0.748
Municipal Health Department	9 (13.2)	45 (66.2)	14 (20.6)		13 (19.1)	35 (51.5)	20 (29.4)		0 (0)	3 (7.7)	36 (92.3)		4 (14.3)	11 (39.3)	13 (46.4)		2 (5.1)	20 (26.6)	27 (69.2)	
Basic Health Unit	16 (11.9)	104 (77.6)	14 (10.5)		24 (18.1)	66 (49.6)	43 (32.3)		0 (0)	15 (18.1)	68 (81.9)		6 (10.5)	25 (43.9)	26 (45.6)		4 (4.8)	23 (27.7)	56 (67.5)	
Mental health specialties	1 (6.7)	9 (60.0)	5 (33.3)		3 (20.0)	6 (40.0)	6 (40.0)		0 (0)	1 (8.3)	11 (91.7)		0 (0)	0 (0)	3 (100.0)		1 (8.3)	0 (0)	11 (91.7)	
Others	4 (17.4)	16 (69.6)	3 (13.0)		6 (26.1)	9 (39.1)	8 (34.8)		1 (5.9)	3 (17.6)	13 (76.5)		1 (16.7)	1 (16.7)	4 (66.6)		1 (5.9)	4 (23.5)	12 (70.6)	
Length of time in position																				
< 1 year	6 (7.4)	64 (79.0)	11 (13.6)	0.003	21 (25.9)	42 (51.9)	18 (22.2)	0.056	0 (0)	6 (12.5)	42 (87.5)	0.499	3 (8.1)	16 (43.2)	18 (48.7)	0.59	3 (6.2)	8 (16.7)	37 (77.1)	0.218
≥ 1 year and < 5 years	30 (18.4)	116 (71.2)	17 (10.4)		34 (21.0)	82 (50.6)	46 (28.4)		1 (1.0)	20 (19.6)	81 (79.4)		9 (13.3)	23 (33.8)	36 (52.9)		7 (6.9)	28 (27.4)	67 (65.7)	
≥ 5 years	5 (6.3)	55 (69.6)	19 (24.1)		13 (16.5)	32 (40.5)	34 (43.0)		0 (0)	5 (10.9)	41 (89.1)		2 (5.6)	12 (33.3)	22 (61.1)		0 (0)	14 (30.4)	32 (69.6)	

Physical Education, Nutrition, Speech Therapy, Occupational Therapy, Physiotherapy, Pharmacology, Administration, and related areas.

Furthermore, the level of education was associated with knowledge in “Essential care and practices” (p = 0.029), “Suicide and self-mutilation” (p = 0.045), and “Depression” (p = 0.013). In this context, participants with *stricto sensu* training (master’s and/or doctorate) seemed to have greater knowledge about the “Essential care and practices” and “Suicide and self-mutilation” modules, while those with *a lato sensu* graduate degree seemed to have better performance in the “Depression module.”

Furthermore, there was a relationship between longer experience in the position and greater knowledge regarding “Essential care and practices” (p = 0.003).

## DISCUSSION

This study brings relevant contributions on the previous knowledge of mental health of PHC professionals with higher education, who work in regions that implement the PAS methodology and were nominated to participate in the training of MhGAP-IG multipliers.

The main knowledge gaps found, in line with the literature^7,19,20^, were themes related to basic principles of communication, covered in the module “Essential care and practices.” This finding is relevant because, for effective mental health care in PHC, good knowledge of technical topics is not enough, such as a higher performance in the “Depression” module, but it is crucial to articulate this knowledge through adequate communicative interaction with users and the team. It is important to highlight how communication skills are an indispensable requirement in daily teamwork, with a view to interdisciplinarity^21^.

Notably, graduates with a *lato sensu* degree had greater knowledge about depression, while those with a *stricto sensu* degree had a higher percentage of correct answers regarding essential care and practices, as well as suicide and self-mutilation. It is not possible to state that this is a result of training itself, as this study did not identify the area of training, but it is suggested to consider that such a finding may reflect not only training processes, but also the predominant scope of practical activity of professionals with a broad training, who deal with more prevalent issues, such as depression.

Another knowledge gap found is related to the most common presentation of psychological distress in PHC: emotional and physical suffering without diagnostic criteria for a specific disease, which is covered in the “Other important complaints” module^7^. This finding corroborates the statement found in the literature, that professionals who work in PHC come from a disease-centered training model, a legacy of the Cartesian model that still dominates educational and health practices^20^.

When considering experiences in other settings, a systematic review^24^examined the impact of MhGAP-IG in low- and middle-income countries. Among the 33 studies analyzed, five reported the use of the same mhGAP^11^knowledge questionnaire used in this study. In addition to this questionnaire, some also used other assessment approaches, e.g., measures of attitudes towards mental illness (MICA-4 and CAMI), self-efficacy and practice, confidence measures, stigma assessment, clinical competence through the Enhancing Assessment of Common Therapeutic factors tool (Enact), along with focus groups and key informant interviews. Others reported assessing readiness for change, using the Readiness for Change Questionnaire (RCQ)^23^instrument.

In the Brazilian scenario, an exploratory-descriptive study was developed, which discusses the educational format of virtual improvement of a project conducted in Ceará, with the objective of training health professionals through MhGAP-IG. In this context, it was decided to use a questionnaire composed of ten questions per module^24^, in a similar way to this study.

If we want to increase the effectiveness of actions to tackle the most prevalent problems and overcome the fragmented logic of care towards comprehensive care, we need to reinforce the relationship between health determinants, social vulnerability, and complaints of emotional and physical distress^25^ throughout the PAS activities, in order to develop PHC professionals’ skills to work with this type of demand, which is so prevalent at this level of care.

At the international level, authors who researched training for the use of MhGAP-IG point out the need for broader training, one which is not limited only to the technical knowledge of health professionals, but also encourages changes in work processes and investments at other levels of management, including, fundamentally, the federal and regional levels^26,27^.

This recommendation is consistent with the choice to include training in the use of MI-mhGAP across the board in the PAS methodology, with a view to problematizing and reflecting on the role of PHC as a network organizer. Studies on the practical application of PAS converge in highlighting the importance of qualifying, that is, of continually developing the necessary skills in state and municipal managers, as well as in tutors who are directly responsible for supporting professionals and teams in the exercise of their care and management functions^28^.

It is noteworthy that the benefits of this choice are also seen from the inverse perspective, as the change in work processes that PAS aims to achieve must be accompanied by the construction of a solid base of knowledge in the line of care worked^29^and training for the use of MhGAP-IG aims to overcome the gap in mental health knowledge among PHC professionals, recognized by national and international authors^30,31^. The strategy of investing in the qualification of professionals, through education and ongoing training in this area, is highlighted in the literature as the main action for the successful integration of mental health in PHC^32^.

As a limitation of this study, it is worth mentioning the convenience sample which, despite being homogeneous, composed of professionals with higher education in PHC, may result in difficulties in generalizing the findings. There was also difficulty in accessing the collection instrument for some study participants, due to limited internet access.

Although there are several ways to assess the acquisition of knowledge, skills and attitudes in the provision of mental health care, we chose to use the specific WHO instrument to assess knowledge in mental health, as the assessment carried out in this article refers to the preliminary stage of a project that foresees the subsequent use of the mhGAP intervention guide for the training of multipliers and PHC professionals. The strengths of the study include the participation of health professionals from three different regions of Brazil: North, Northeast, and Midwest.

As future perspectives, there is the planning, execution, and monitoring of multiplication (ToHP) carried out with health professionals, which will be agreed according to the local realities of the health regions, with the support of state and municipal health departments, as well as the technical team that integrates the project.

In addition, the “Mental Health in PHC*”* project team will make the standardized material needed to carry out the ToHP available on the accompanying website (e-Planifica), such as power point presentations, videos and scripts for dramatizations, roleplays and personal stories. An mhGAP Multiplier Guide was worked out (https://planificasus.com.br/arquivo- download.php?hash=fb4e2d6527f589820e6a408ebf4481c847e16cdb&t=1685538346&type =biblioteca), in which information is gathered to support them in organizing and conducting multiplication.

## CONCLUSION

This article makes practical contributions, as it identified which knowledge topics are most fragile in the mhGAP training multipliers, thus enabling them to intensify their approach during the thematic operational stages of the PAS. The results show that, in addition to technical knowledge about mental health, it is necessary to develop communication skills in everyday teamwork. In this sense, we suggest the need to articulate knowledge, through communicative interaction between users and the team, in order to overcome the knowledge gap identified and make PHC professionals able to provide mental health care at this level of care, sharing the care of the most serious cases with the other points of the Psychosocial Care Network in a qualified manner.
