# Supplementary material for: The knowledge of primary health care professionals regarding mental health: diagnosis by mhGAP
Source: Rev Saude Publica. 2024 Apr 1;57(Suppl 3):4s. doi: 10.11606/s1518-8787.2023057005272 (PMC11037900; doi:10.11606/s1518-8787.2023057005272)
Supplement: Translation

# Translation: Conhecimento de profissionais da atenção primária em saúde mental: diagnóstico pelo mhGAP

**Authors:** Joana Moscoso Teixeira de Mendonça, Ilana Eshriqui, Leticia Yamawaka de Almeida, Valmir Vanderlei Gomes, Lívia Schunk, Ana Alice Freire de Sousa, Larissa Karollyne de Oliveira Santos, Sandra Fortes

**Affiliations:** I Brasil Hospital Israelita Albert Einstein. Centro de Estudos, Pesquisa e Prática em Atenção Primária à Saúde e Redes (CEPPAR). São Paulo, SP, Brasil; II Brasil Universidade do Estado do Rio de Janeiro. Departamento de Especialidades Médicas. Rio de Janeiro, RJ, Brasil

**Keywords:** Saúde Mental, Atenção Primária à Saúde, Capacitação de Recursos Humanos em Saúde

## Abstract

**OBJETIVO:**

Analisar o conhecimento sobre temas prioritários do cuidado em saúde mental de atores estratégicos que atuam em regiões nas quais se utiliza a metodologia da Planificação da Atenção à Saúde (PAS).

**MÉTODOS:**

Trata-se de um estudo quantitativo, descritivo, transversal e observacional realizado com profissionais de seis regiões de saúde, distribuídas em três estados brasileiros (Goiás, Rondônia e Maranhão) e vinculadas ao projeto “Saúde mental na APS*”* do Programa de Apoio ao Desenvolvimento Institucional do Sistema Único de Saúde (Proadi-SUS). A amostra foi composta por profissionais que participaram da etapa de formação de multiplicadores do manual de intervenções para transtornos mentais, neurológicos e por uso de álcool e outras drogas na rede de atenção básica à saúde, no período de julho a setembro de 2022. A coleta de dados foi realizada a partir de um instrumento autoaplicável, em formato eletrônico, composto por um bloco com itens socioeconômicos e um questionário estruturado para avaliação do conhecimento dos participantes acerca de temas prioritários em saúde mental. Para análise dos dados, foram empreendidas análises descritivas e comparação de proporções.

**RESULTADOS:**

Participaram do estudo 354 profissionais de saúde. Em relação ao percentual de acerto no questionário sobre temas prioritários em saúde mental, as medianas mais altas foram identificadas no módulo de “Depressão”. Em contrapartida, o conteúdo referente aos módulos “Cuidados e práticas essenciais” e “Outras queixas importantes” apresentaram os valores mais baixos. Além disso, identificou-se que algumas características dos participantes apresentaram associações com o percentual de acertos nos módulos do questionário.

**CONCLUSÕES:**

Os achados revelam oportunidades de melhoria, principalmente no conhecimento relacionado às habilidades de comunicação e a abordagem ao sofrimento emocional e físico sem critérios diagnósticos para uma doença específica, oferecendo subsídios para o planejamento de ações que visem a intensificação do olhar destas temáticas durante as etapas operacionais da PAS.

## INTRODUÇÃO

No âmbito do cuidado em saúde mental, a crescente carga global dos transtornos mentais^1^ associada ao seu grande ônus econômico^2,3^ e à escassez de recursos humanos com conhecimentos adequados em saúde mental são elementos de um cenário relacionado à lacuna entre a necessidade de cuidado e a oferta de tratamento minimamente efetivo para essas condições^4,5^.

A proposta para o enfrentamento desta lacuna preconiza, entre outros aspectos, o estabelecimento de uma rede efetiva de cuidados que contemple um conjunto diversificado de serviços, uma vez que apenas aqueles especializados em saúde mental não garantirão a integralidade da assistência^6^.

Assim, os serviços de atenção primária à saúde (APS) se destacam como estratégicos na oferta de cuidado em saúde mental especialmente por dois pontos: a prevalência dos transtornos mentais^7^ e o papel fundamental da APS no enfrentamento de condições sanitárias com alta prevalência; e a integralidade do cuidado, enquanto atributo da APS^8^– uma vez que não se pode oferecer um cuidado integral e negligenciar as questões de saúde mental^9^.

Nesse sentido, o Programa de Ação para Lacunas de Saúde Mental (*mental health Gap Action Programme* – mhGAP, na sigla em inglês), lançado pela Organização Mundial de Saúde (OMS) em 2008, busca responder à pergunta: “o que pode ser feito na assistência à saúde de rotina, por profissionais de saúde não especializados, para tratar pessoas portadoras de transtornos mentais?”^10^.

Entre os desdobramentos desse plano de ação, a OMS elaborou a primeira versão do Manual de Intervenções mhGAP (MI-mhGAP), que é atualizada periodicamente com o objetivo de apoiar a implementação das diretrizes propostas, a partir da capacitação de profissionais não especialistas da APS. A versão 2.0 do MI-mhGAP^11^se caracteriza como uma ferramenta técnica, de baixo custo e alto impacto, que apresenta o manejo integrado dos transtornos mentais, neurológicos e por uso de álcool e outras drogas, por meio do uso de protocolos para a decisão clínica.

Ressalta-se que, além de conhecimentos técnicos, o trabalho em saúde mental na APS exige dos profissionais o conhecimento sobre as políticas públicas de saúde, o território, o perfil epidemiológico da população e a rede de cuidados, assim como são fundamentais as habilidades para acolher, ouvir, comunicar-se e trabalhar em equipe^12^.

Dessa forma, o Projeto do Programa de Apoio ao Desenvolvimento Institucional do Sistema Único de Saúde (Proadi-SUS), intitulado “Saúde mental na APS”, une a estratégia de capacitação do uso do MI-mhGAP para avaliação manejo e seguimento dos temas prioritários em saúde mental na APS à metodologia da Planificação da Atenção à Saúde (PAS). Trata-se de uma metodologia que permite aperfeiçoar a competência das equipes de saúde para o planejamento, a organização e o monitoramento de processos de trabalho, com foco na necessidade dos usuários sob sua responsabilidade^13^.

O processo se ancora num robusto componente pedagógico, com vistas ao desenvolvimento de conhecimentos, habilidades e atitudes em trabalhadores e gestores para ofertar uma atenção à saúde de qualidade e que agregue valor ao usuário^14^. Seu objetivo é desenvolver as equipes para adequada operacionalização das Redes de Atenção à Saúde (RAS), com ênfase em diversas linhas de cuidado prioritárias. Essa união visa fortalecer a linha de cuidado em saúde mental, na medida em que qualifica a APS para desempenhar seu papel de organizadora e coordenadora do cuidado em saúde na RAS.

Diante desse contexto, entende-se que para o desenvolvimento de estratégias de organização e qualificação do cuidado em saúde mental é necessário o diagnóstico do nível de conhecimento prévio dos profissionais na temática. Assim, este estudo teve como objetivo analisar o conhecimento sobre temas prioritários do cuidado em saúde mental de profissionais de nível superior da APS que atuam em regiões que executam a metodologia da PAS e foram indicados para serem multiplicadores do treinamento para uso do MI-mhGAP.

## MÉTODO

### Cenário do Estudo

O estudo foi realizado nas regiões de saúde que participam do Projeto Proadi-SUS “Saúde mental na APS*”*, a saber: três no estado de Goiás, duas em Rondônia e uma no Maranhão. As regiões foram selecionadas de acordo com três critérios prioritários: 1). experiência prévia de no mínimo dois anos em implantação da PAS; 2) macroprocessos da “Construção social da APS”, com ciclos de melhoria iniciados (ex.: diagnóstico situacional, territorialização, cadastro familiar, entre outros); e 3) abrangência de municípios executando a PAS. Sinaliza-se que o projeto “Saúde mental na APS*”* não tem como objetivo implantar a PAS nessas regiões, mas qualificar os processos já implantados (ou em implantação), com foco nas questões relacionadas ao cuidado em saúde mental.

A Figura apresenta o desenho operacional do projeto “Saúde mental na APS”, no qual é realizada a capacitação de multiplicadores e profissionais para uso do MI-mhGAP, em processo transversal à execução da PAS.

**Figura:** Desenho operacional do projeto “Saúde mental na APS”.

APS: atenção primária à saúde; RAPS: Rede de Atenção Psicossocial; ToHP: Training of health professionals; ToTS: Training of trainers and supervisor; MI-mhGAP: Manual de Intervenções mhGAP (mental health Gap Action Programme).

Conforme pode ser observado, no contexto da PAS são realizadas ações de gerenciamento e processo de tutoria, à luz de ciclos de melhoria contínua *Plan-Do-Study-Act* (PDSA)^15^, a fim de qualificar os processos de trabalho ao longo das temáticas abordadas nas quatro etapas operacionais. Por meio do gerenciamento da PAS, promove-se apoio ao corpo técnico das Secretarias Estaduais de Saúde (SES) e Secretarias Municipais de Saúde (SMS) para tomada de decisão ao longo das etapas preparatórias, operacionais e de controle. A partir do processo de tutoria são realizados momentos de alinhamento teórico conceitual, reflexão, planejamento e execução de melhorias, para que se efetivem as mudanças no *modus operandi* das equipes e dos serviços^16^.

Transversalmente à PAS, são realizados os treinamentos para o uso do MI-mhGAP. Esta etapa é organizada em dois momentos, conforme preconizado pela OMS ^17^:

- Treino dos profissionais de saúde (tradução do termo em inglês: *Training of health professionals* – ToHP), que se configura em um treinamento que busca fomentar o desenvolvimento de competências essenciais para o cuidado às pessoas com condições MNS (que, na presente proposta, envolvem os módulos práticas e cuidados essenciais; depressão, autoagressão e suicídio, psicose, outras queixas significativas em saúde mental e uso problemático de álcool)^17^;
- Treino de treinadores e supervisores (tradução do termo em inglês: *Training of trainers and supervisor* – ToTS), que visa preparar os profissionais (multiplicadores) responsáveis pela replicação do conteúdo em suas regiões, a partir da apresentação de propostas e modelos de ensino-aprendizagem de adultos^17^.

Ao se tratar especificamente do treinamento dos multiplicadores, a formação possui carga horária de 40h, distribuídas entre as atividades do ToHP e ToTS. Com o objetivo de facilitar a organização da multiplicação nos diferentes territórios, os participantes foram distribuídos em duas turmas. A turma “A” realizou o treinamento dos módulos de “Depressão” e “Autoagressão e Suicídio” e a turma “B” os módulos de “Psicose” e “Uso problemático de álcool”.

Além disso, os módulos “Cuidados e práticas essenciais” e “Outras queixas importantes” foram abordados em ambas as turmas, por se compreender que tratam de aspectos básicos do cuidado e são a rotina do trabalho em saúde mental na APS. Ao término da formação, orientou-se a formação de duplas com representantes das turmas A e B, para conduzir a multiplicação dos seis módulos do TOHP aos demais profissionais da APS dos municípios das regiões participantes.

### Desenho do Estudo

Trata-se de um estudo quantitativo, descritivo, transversal, observacional aprovado pela Comitê de Ética em Pesquisa do Hospital Israelita Albert Einstein (número do parecer: CAAE 12395919000000071).

### População e Amostra Estudo

A amostragem foi realizada por conveniência, considerando os profissionais de saúde indicados para participar do treinamento de multiplicadores do MI-mhGAP. A indicação foi realizada a partir dos seguintes critérios, sugeridos pela equipe do projeto: formação de nível superior na área da saúde; experiência profissional na área de saúde mental na APS; atuação nas secretarias estaduais, municipais ou nos serviços de saúde das regiões supracitadas, apresentar habilidades pedagógicas e de comunicação e ter disponibilidade de carga horária para realizar a multiplicação da formação nas regiões.

Assim, para este estudo, foram considerados elegíveis os 366 profissionais que participaram do treinamento de multiplicadores no período de julho a setembro de 2022. Destes, 197 concluíram as atividades na turma “A”, 125 na turma “B” e 44 realizaram o treinamento em ambas as turmas. Sinaliza-se que, entre os profissionais elegíveis, somente 83 mencionaram ter formação em saúde mental.

### Recrutamento e Coleta de Dados

A abordagem aos profissionais foi realizada conforme o cronograma das turmas de formação dos multiplicadores (julho a setembro de 2022), de modo que o convite para participação do estudo e a coleta de dados foi realizada antes do início do treinamento. Na ocasião, foram apresentados os principais aspectos da pesquisa e realizado o convite para participação do estudo. O Termo de Consentimento Livre e Esclarecido foi disponibilizado em formato eletrônico e aqueles que manifestaram interesse em participar do estudo, após a confirmação do aceite no documento, eram direcionados a um instrumento estruturado autoaplicável, composto por um bloco com itens sociodemográficos e profissionais; seguido pelo questionário do mhGAP^17^para avaliação do conhecimento dos participantes acerca de temas prioritários em saúde mental, traduzido para o português pela equipe responsável pelo treinamento na Universidade Estadual do Rio de Janeiro. O questionário é composto por 50 questões de múltipla escolha, distribuídas em cinco módulos de avaliação, os quais foram aplicados conforme previsto nas turmas A e/ou B (Quadro). Destaca-se que o treinamento do módulo “Uso problemático de álcool” foi adaptado para a cultura brasileira e, por isto, optou-se por não considerar os itens referentes a esta temática no questionário. O tempo programado para resposta do questionário foi de 20 minutos.

**Quadro:** Descrição do conteúdo abordado no questionário estruturado (julho a setembro de 2022).

Módulos	Número de Itens do questionário	Turmas
Cuidados e Práticas Essenciais	15 itens	A e B
Outras Queixas Importantes	08 itens	A e B
Depressão	10 itens	A
Autoagressão/Suicídio	8 itens	A
Psicose	9 itens	B
Uso Problemático de Álcool	Não avaliado	B

Os dados do estudo foram coletados e gerenciados pela ferramenta eletrônica *Research Electronic Data Capture* (REDcap)^18^, que se trata de uma plataforma para coleta, armazenamento e gerenciamento de dados de pesquisas. Dessa forma, o risco de perda de confidencialidade é mínimo, uma vez que os dados são hospedados e gerenciados apenas pelo acesso institucional à plataforma Redcap por meio de senha com sistema de proteção de dupla camada (login e senha).

### Análise dos Dados

As respostas corretas foram codificadas como 1 e as incorretas como 0. A partir disso, foram calculadas as notas em cada módulo, que foram obtidas a partir da somatória e cálculo dos percentuais de acertos por módulo da formação de multiplicadores. Os percentuais de acertos foram descritos em mediana e intervalo interquartil e categorizados em < 50%; 50–75%; ≥ 75% dos acertos. Foram descritas frequências e proporções das características dos participantes, assim como dos percentuais de acerto. Utilizou-se teste qui-quadrado para comparar as proporções das categorias de acerto segundo características dos participantes. Adotou-se p-valor < 0,05 para identificar associações estatisticamente significantes. O software Stata (versão 12, 2011, StataCorp LP) foi utilizado para análise dos dados.

## RESULTADOS

Entre os 366 participantes da formação de multiplicadores, 354 (96,7%) aceitaram participar da pesquisa. A Tabela 1 descreve as características dos participantes, os quais apresentavam em média 37,6 anos (DP = 10,1) e, em sua maioria, eram do sexo feminino (83,7%) e referiram cor de pele parda (57,6%). Observou-se o predomínio de profissionais com formação em enfermagem (50,5%), pós-graduação *lato sensu* (57,3%), que atuavam há mais de um ano no cargo atual (76,5%) e trabalhavam em unidade básica de saúde (UBS) (41,0%).

**Tabela 1:** Perfil sociodemográfico e profissional dos participantes (julho a setembro de 2022).

Características	n (%)
Turmas (n = 354)	
A	197 (55,6)
B	138 (39,0)
A e B	19 (5,4)
Sexo (n = 345)	
Feminino	289 (83,7)
Masculino	56 (16,2)
Raça/cor (n = 342)	
Branca	123 (36,0)
Parda	197 (57,6)
Preta	21 (6,1)
Amarela	1 (0,3)
Categoria profissional (n = 305)	
Médico	21 (7,8)
Enfermeiro	154 (50,5)
Psicólogo	69 (22,6)
Cirurgião dentista	12 (3,9)
Assistente social	10 (3,3)
Outras^a^	36 (11,8)
Nível de escolaridade (n = 342)	
Graduação	116 (33,9)
Pós-graduação *lato sensu*	196 (57,3)
Pós-graduação *stricto sensu*	30 (8,8)
Local de atuação (n = 344)	
Secretaria Estadual de Saúde	90 (26,2)
Secretaria Municipal de Saúde	73 (21,2)
Unidade básica de saúde	141 (41,0)
Especialidade em saúde mental	16 (4,6)
Outros	24 (7,0)
Tempo de atuação no cargo (n = 344)	
< 1 ano	81 (23,5)
≥ 1 ano e < 5 anos	174 (50,6)
≥ 5 anos	89 (25,9)
Estado em que realizou o treinamento (n = 339)	
Goiás (GO)	169 (49,8)
Maranhão (MA)	68 (20,1)
Rondônia (RO)	102 (30,1)

^a^ Categorias profissionais com proporção menor que 3% (Educação Física, Nutrição, Fonoaudiologia, Terapia Ocupacional, Fisioterapia, Farmacologia, Administração e áreas correlatas).

Em relação ao conhecimento prévio em temas prioritários de saúde mental, avaliados pelos questionários, a mediana mais alta dos percentuais de acerto foi identificada no módulo “Depressão”. Em contrapartida, o conteúdo referente aos módulos “Cuidados e práticas essenciais” e “Outras queixas importantes” apresentou os valores mais baixos (Tabela 2).

**Tabela 2:** Descrição do percentual de acerto nos módulos oferecidos no treinamento (julho a setembro de 2022).

Módulo	Mediana (IQQ)	% de acertos		

		< 50%	≥ 50 e < 75%	≥ 75%
		
		n (%)	n (%)	n (%)
Cuidados e práticas essenciais (n = 328)	66,7(53,3–73,3)	44 (13,4)	236 (72,0)	48 (14,6)
Depressão (n = 199)	90,0 (80,0–100,0)	1(0,5)	31(15,6)	167(83,9)
Psicose (n = 143)	77,8 (66,7–88,9)	14 (9,8)	52 (36,4)	77(53,8)
Suicídio e automutilação (n = 199)	75,0 (62,5–87,5)	10 (5,0)	52 (26,1)	137(68,9)
Outras queixas importantes (n = 325)	62,5 (50,0–75,0)	69 (21,2)	157 (48,3)	99(30,5)

IQQ: Intervalo Inter quartil.

A Tabela 3 apresenta a descrição do percentual de acerto nos módulos do treinamento, de acordo com as características dos participantes. Identificou-se que a variável categoria profissional apresentou associação com o conhecimento referente aos módulos de “Cuidados e práticas essenciais” (p = 0,004), “Outras queixas importantes” (p = 0,033) e “Suicídio e automutilação” (p = 0,044), de modo que médicos pareceram ter melhor desempenho nos módulos “Cuidados e práticas essenciais” e “Outras queixas importantes”; e psicólogos e cirurgiões dentistas nos módulos de “Suicídio e automutilação”.

**Tabela 3:** Descrição do percentual de acertos nos módulos do treinamento, de acordo com as características dos participantes (julho a setembro de 2022).

Características	Cuidados e práticas essenciais				Outras queixas importantes				Depressão				Psicose				Suicídio e automutilação			
				
	% de acertos – n (%)			valor de p	% de acertos – n (%)			valor de p	% de acertos – n (%)			valor de p	% de acertos – n (%)			valor de p	% de acertos – n (%)			valor de p
				
	< 50%	≥ 50 e < 75%	≥ 75%		< 50%	≥ 50 e < 75%	≥ 75%		< 50%	≥ 50 e < 75%	≥ 75%		< 50%	≥ 50 e < 75%	≥ 75%		< 50%	≥ 50 e < 75%	≥ 75%	
Categoria profissional																				
Médico	2 (8,3)	14 (58,3)	8 (33,3)	0,004	5 (20,8)	6 (25,0)	13 (54,2)	0,033	1 (7,1)	2 (14,3)	11 (78,6)	0,064	1 (10,0)	2 (20,0)	7 (70,0)	0,823	2 (14,3)	2 (14,3)	10 (71,4)	0,044
Enfermeiro	15 (10,3)	115 (79,3)	15 (10,3)		35 (24,3)	73 (50,7)	36 (25,0)		0 (0)	19 (20,4)	74 (79,6)		6 (10,3)	23 (39,7)	29 (50,0)		4 (4,3)	31 (33,3)	58 (62,4)	
Psicólogo	5 (7,8)	48 (75,0)	11 (17,2)		8 (12,5)	35 (54,7)	21 (32,8)		0 (0)	2 (4,9)	39 (95,1)		2 (6,9)	9 (31,0)	18 (62,1)		0 (0)	5 (12,2)	36 (87,8)	
Cirurgião dentista	3 (25,0)	6 (50,0)	3 (25,0)		4 (33,3)	4 (33,3)	4 (33,3)		0 (0)	1 (14,3)	6 (85,7)		1 (16,7)	1 (16,7)	4 (66,7)		0 (0)	1 (14,3)	6 (85,7)	
Assistente social	1 (11,1)	7 (77,8)	1 (11,1)		0 (0)	5 (55,6)	4 (44,4)		0 (0)	1 (20,0)	4 (80,0)		0 (0)	1 (25,0)	3 (75,0)		1 (20,0)	1 (20,0)	3 (60,0)	
Outras^a^	11 (31,4)	19 (54,3)	5 (14,3)		12 (34,3)	15 (42,9)	8 (22,8)		0 (0)	1 (16,7)	20 (80,3)		2 (18,2)	5 (45,4)	4 (36,4)		3 (12,5)	7 (29,2)	14 (58,3)	
Nível de escolaridade																				
Graduação	18 (16,7)	83 (76,8)	7 (6,5)	0,029	30 (27,8)	52 (48,1)	26 (24,1)	0,079	0 (0)	14 (20,3)	55 (79,7)	0,013	4 (9,5)	16 (38,1)	22 (52,4)	0,714	6 (8,7)	21 (30,4)	42 (60,9)	0,045
Pós-graduação *lato sensu*	20 (10,8)	132 (71,4)	33 (17,8)		33 (17,9)	94 (51,1)	57 (31,0)		0 (0)	15 (13,8)	94 (86,2)		10 (11,6)	29 (33,7)	47 (54,7)		3 (2,8)	30 (27,5)	76 (69,7)	
Pós-graduação *stricto sensu*	3 (10,7)	18 (64,3)	7 (25,0)		4 (14,3)	11 (39,3)	13 (46,4)		1 (6,2)	2 (12,5)	13 (81,3)		0 (0)	6 (46,1)	7 (53,9)		1 (6,3)	0 (0)	15 (93,7)	
Local de atuação																				
Secretaria Estadual de Saúde	11 (13,4)	60 (73,2)	11 (13,4)	0,332	22 (26,8)	40 (48,8)	20 (24,4)	0,776	0 (0)	9 (20,0)	36 (80,0)	0,081	3 (6,5)	14 (30,4)	29 (63,1)	0,445	2 (4,4)	14 (31,1)	29 (64,4)	0,748
Secretaria Municipal de Saúde	9 (13,2)	45 (66,2)	14 (20,6)		13 (19,1)	35 (51,5)	20 (29,4)		0 (0)	3 (7,7)	36 (92,3)		4 (14,3)	11 (39,3)	13 (46,4)		2 (5,1)	20 (26,6)	27 (69,2)	
Unidade Básica de Saúde	16 (11,9)	104 (77,6)	14 (10,5)		24 (18,1)	66 (49,6)	43 (32,3)		0 (0)	15 (18,1)	68 (81,9)		6 (10,5)	25 (43,9)	26 (45,6)		4 (4,8)	23 (27,7)	56 (67,5)	
Especialidades em saúde mental	1 (6,7)	9 (60,0)	5 (33,3)		3 (20,0)	6 (40,0)	6 (40,0)		0 (0)	1 (8,3)	11 (91,7)		0 (0)	0 (0)	3 (100,0)		1 (8,3)	0 (0)	11 (91,7)	
Outros	4 (17,4)	16 (69,6)	3 (13,0)		6 (26,1)	9 (39,1)	8 (34,8)		1 (5,9)	3 (17,6)	13 (76,5)		1 (16,7)	1 (16,7)	4 (66,6)		1 (5,9)	4 (23,5)	12 (70,6)	
Tempo de atuação no cargo																				
< 1 ano	6 (7,4)	64 (79,0)	11 (13,6)	0,003	21 (25,9)	42 (51,9)	18 (22,2)	0,056	0 (0)	6 (12,5)	42 (87,5)	0,499	3 (8,1)	16 (43,2)	18 (48,7)	0,59	3 (6,2)	8 (16,7)	37 (77,1)	0,218
≥ 1 ano e < 5 anos	30 (18,4)	116 (71,2)	17 (10,4)		34 (21,0)	82 (50,6)	46 (28,4)		1 (1,0)	20 (19,6)	81 (79,4)		9 (13,3)	23 (33,8)	36 (52,9)		7 (6,9)	28 (27,4)	67 (65,7)	
≥ 5 anos	5 (6,3)	55 (69,6)	19 (24,1)		13 (16,5)	32 (40,5)	34 (43,0)		0 (0)	5 (10,9)	41 (89,1)		2 (5,6)	12 (33,3)	22 (61,1)		0 (0)	14 (30,4)	32 (69,6)	

^a^Educação Física, Nutrição, Fonoaudiologia, Terapia Ocupacional, Fisioterapia, Farmacologia, Administração e áreas correlatas.

Além disso, o nível de escolaridade teve associação com o conhecimento em “Cuidados e práticas essenciais” (p = 0,029), “Suicídio e automutilação” (p = 0,045) e “Depressão” (p = 0,013). Nesse contexto, participantes com formação *stricto sensu* (mestrado e/ou doutorado) pareceram apresentar maior conhecimento sobre os módulos “Cuidados e práticas essenciais” e “Suicídio e automutilação”, enquanto aqueles com pós-graduação *lato sensu* pareceram ter melhor desempenho no módulo de “Depressão”.

Acrescenta-se, ainda, a relação entre o maior tempo de atuação no cargo e o maior conhecimento frente aos “Cuidados e práticas essenciais” (p = 0,003).

## DISCUSSÃO

Este estudo traz contribuições relevantes sobre o conhecimento prévio em saúde mental de profissionais de nível superior da APS, que atuam em regiões que executam a metodologia da PAS e foram indicados para participar do treinamento de multiplicadores do MI-mhGAP.

Foram encontradas entre as principais lacunas de conhecimento, em consonância com a literatura^7,19,20^, os temas relacionados a princípios básicos de comunicação, abordados no módulo “Cuidados e práticas essenciais”. Esse achado é relevante pois, para um cuidado efetivo em saúde mental na APS, não basta um bom conhecimento em temas técnicos, como a maior performance no módulo “Depressão”, mas é primordial a articulação desses saberes por meio de uma interação comunicativa adequada com os usuários e com a equipe. É importante destacar como as habilidades de comunicação são um requisito indispensável no cotidiano do trabalho em equipe, com vistas à interdisciplinaridade^21^.

Vale ressaltar que os profissionais com pós-graduação *lato sensu* apresentaram maior conhecimento sobre depressão, enquanto os profissionais com pós-graduação *stricto sensu* apresentaram maior percentual de acerto sobre os cuidados e práticas essenciais, bem como suicídio e automutilação. Não é possível afirmar que se trata da formação em si, pois este estudo não identificou a área da formação, mas sugere-se considerar que tal achado possa refletir não apenas processos formativos, mas também o escopo de atuação prática predominante dos profissionais com formação *lato sensu*, que lidam com questões mais prevalentes, à exemplo da depressão.

Outra lacuna de conhecimento encontrada está relacionada a apresentação mais comum do sofrimento psíquico na APS: o sofrimento emocional e físico sem critérios diagnósticos para uma doença específica, que é abordado no módulo “Outras queixas importantes”^7^. Esse achado corrobora a afirmação encontrada na literatura, de que os profissionais que atuam na APS vêm de um modelo de formação centrado na doença, herança do modelo cartesiano que ainda domina as práticas educativas e de saúde^20^.

Ao se considerar experiências em outros cenários, uma revisão sistemática^24^examinou o impacto do MI-mhGAP em países de baixa e média renda. Entre os 33 estudos analisados, cinco reportaram a utilização do mesmo questionário de conhecimento do mhGAP^11^ usado neste estudo. Além deste questionário, alguns utilizaram também outras abordagens de avaliação, por exemplo, medidas de atitudes em relação à doença mental (MICA-4 e CAMI), autoeficácia e prática, medidas de confiança, avaliação de estigma, competência clínica por meio do *Enhancing Assessment of Common Therapeutic factors tool* (Enact), juntamente com grupos focais e entrevistas com informantes-chave. Outros reportaram a avaliação de prontidão para mudanças, utilizando o instrumento *Readiness for Change Questionnaire* (RCQ)^23^.

No cenário brasileiro, desenvolveu-se um estudo exploratório-descritivo, que discute o formato educacional de aperfeiçoamento virtual de um projeto conduzido no Ceará, com o objetivo de capacitar profissionais de saúde por meio do MI-mhGAP. Nesse contexto, optou-se por utilizar um questionário composto por dez questões por módulo^24^, de forma similar a este estudo.

Se quisermos aumentar a resolutividade das ações frente aos problemas mais prevalentes e superar a lógica fragmentada de cuidado em direção ao cuidado integral, devemos reforçar, ao longo das atividades da PAS, a relação entre os determinantes em saúde, a vulnerabilidade social e as queixas de sofrimento emocional e físico^25^, a fim de desenvolver, nos profissionais da APS, as competências para trabalhar com esse tipo de demanda tão prevalente nesse nível de atenção.

No âmbito internacional, autores que pesquisaram a capacitação para o uso do MI-mhGAP apontam que uma formação mais ampla, que não se limite apenas ao conhecimento técnico dos profissionais de saúde, mas também fomente mudanças em processos de trabalho e investimentos em outros níveis de gestão, incluindo, fundamentalmente, os níveis federal e regional^26,27^.

Essa recomendação é consistente com a escolha de inserir o treinamento para uso do MI-mhGAP de forma transversal à metodologia da PAS, visando problematizar e refletir sobre o papel da APS como ordenadora da rede. Estudos acerca da aplicação prática da PAS convergem ao ressaltar a importância de qualificar, ou seja, de desenvolver continuamente as competências necessárias nos gestores estaduais e municipais, bem como nos tutores que ficam diretamente responsáveis por apoiar profissionais e equipes no exercício de suas funções assistenciais e gerenciais^28^.

Ressalta-se que os benefícios dessa escolha também são verificados na perspectiva inversa, na medida em que a mudança dos processos de trabalho que a PAS visa alcançar deve ser acompanhada pela construção de uma base sólida de conhecimento na linha de cuidado trabalhada^29^e a capacitação para o uso do MI-mhGAP visa superar a lacuna de conhecimento em saúde mental pelos profissionais da APS, reconhecida por autores nacionais e internacionais^30,31^. A estratégia de investimento na qualificação dos profissionais, por meio de educação e capacitação permanente nesta área, é ressaltada na literatura como a principal ação para o sucesso da integração da saúde mental na APS^32^.

Como limitação deste estudo, cabe mencionar a amostra de conveniência que, apesar de ser homogênea, ao ser composta por profissionais de nível superior da APS, pode implicar em dificuldades de generalizar os achados. Houve ainda dificuldade de acesso ao instrumento de coleta por alguns participantes do estudo, devido à limitação de acesso à internet.

Apesar de existirem diversas formas de avaliar a aquisição de conhecimentos, habilidades e atitudes na prestação de cuidados em saúde mental, optou-se por utilizar o instrumento específico da OMS para avaliar conhecimentos em saúde mental, pois a avaliação realizada neste artigo se refere à etapa preliminar de um projeto que prevê a posterior utilização do manual de intervenção do mhGAP para o treinamento de multiplicadores e profissionais da APS. Destaca-se como pontos fortes do estudo a participação de profissionais de saúde oriundos de três regiões diferentes do Brasil: Norte, Nordeste e Centro-Oeste.

Como perspectivas futuras, tem-se o planejamento, execução e monitoramento da multiplicação (ToHP) realizada com os profissionais de saúde, que será pactuado de acordo com as realidades locais das regiões de saúde, com apoio das secretarias estaduais e municipais de saúde, bem como da equipe técnica que integra o projeto.

Além disso, a equipe do projeto “Saúde mental na APS*”* disponibilizará no site de acompanhamento (e-Planifica) o material padronizado necessário para a realização do ToHP, como apresentações de *power point*, vídeos e roteiros de dramatização, *roleplays* e histórias pessoais. Foi elaborado um Manual do Multiplicador mhGAP (https://planificasus.com.br/arquivo-download.php?hash=fb4e2d6527f589820e6a408ebf4481c847e16cdb&t=1685538346&type=biblioteca), em que são reunidas informações para apoiá-los na organização e condução da multiplicação.

## CONCLUSÃO

Este artigo traz contribuições práticas, uma vez que identificou quais os temas de conhecimento mais frágeis nos multiplicadores da capacitação mhGAP, permitindo, assim, intensificar a abordagem desses temas durante as etapas operacionais temáticas da PAS. Os resultados permitem concluir que, para além do conhecimento técnico acerca da saúde mental, faz-se necessário desenvolver habilidades de comunicação no cotidiano do trabalho em equipe. Neste sentido, sugere-se a necessidade de articulação de saberes, por meio de interação comunicativa entre usuários e equipe, a fim de superar a lacuna de conhecimento identificada e tornar os profissionais de APS aptos a prestar o cuidado em saúde mental nesse nível de atenção, compartilhando, de forma qualificada, o cuidado dos casos mais graves com os demais pontos da Rede de Atenção Psicossocial.
